# The Application of Molecular Spectroscopy in Combination with Chemometrics for Halal Authentication Analysis: A Review

**DOI:** 10.3390/ijms21145155

**Published:** 2020-07-21

**Authors:** Abdul Rohman, Anjar Windarsih

**Affiliations:** 1 Department of Pharmaceutical Chemistry, Faculty of Pharmacy, Universitas Gadjah Mada, Yogyakarta 55281, Indonesia; 2 Institute of Halal Industry and Systems (IHIS), Universitas Gadjah Mada, Yogyakarta 55281, Indonesia; 3 Research Division for Natural Product Technology (BPTBA), Indonesian Institute of Sciences (LIPI), Yogyakarta 55861, Indonesia; anjarwindarsih2@gmail.com

**Keywords:** authentication, chemometrics, food and pharmaceutical, halal, molecular spectroscopy

## Abstract

Halal is an Arabic term used to describe any components allowed to be used in any products by Muslim communities. Halal food and halal pharmaceuticals are any food and pharmaceuticals which are safe and allowed to be consumed according to Islamic law (Shariah). Currently, in line with halal awareness, some Muslim countries such as Indonesia, Malaysia, and Middle East regions have developed some standards and regulations on halal products and halal certification. Among non-halal components, the presence of pig derivatives (lard, pork, and porcine gelatin) along with other non-halal meats (rat meat, wild boar meat, and dog meat) is typically found in food and pharmaceutical products. This review updates the recent application of molecular spectroscopy, including ultraviolet-visible, infrared, Raman, and nuclear magnetic resonance (NMR) spectroscopies, in combination with chemometrics of multivariate analysis, for analysis of non-halal components in food and pharmaceutical products. The combination of molecular spectroscopic-based techniques and chemometrics offers fast and reliable methods for screening the presence of non-halal components of pig derivatives and non-halal meats in food and pharmaceutical products.

## 1. Introduction

Halal is an Arabic term used to describe any products which are allowed to be consumed by Muslims, according to Shariah (Islamic law) [1]. Muslim societies are not allowed to consume any products containing non-halal components, except under extremely exceptional conditions [2]. Halal products, including food and pharmaceuticals, are defined as any products including food, cosmetics, and pharmaceuticals that contain ingredients permitted under Shariah law that fulfill the following conditions: (a) do not contain any parts or products of animals that are non-halal by Shariah law or any parts or products of animals which are not slaughtered according to Shariah law; (b) do not contain najs (animals such as amphibians, pig and its derivatives, blood and carrions); (c) safe for human use, e.g., non-poisonous, non-intoxicating or non-hazardous to health according to prescribed dosage; (d) not prepared, processed, or manufactured using equipment contaminated with najs; (e) do not contain any human parts or its derivatives that are not permitted by Shariah law; (f) during its preparation, processing, handling, packaging, storage and distribution, the halal pharmaceutical products are physically separated from any other pharmaceutical products that do not meet the requirements stated in items (a), (b), (c), (d), and (e) above, or any other items that have been decreed as non-halal and najs by Shariah law [3].

According to the Holy Quran and some hadith of Prophet Muhammad, non-halal components include carrion or dead animals, blood (flowing or congealed), pig derivatives, animals that are not slaughtered in compliance with Shariah law, animals that are killed accidentally or on purpose through means such as strangling or beating, all types of intoxicants such as alcohol and drugs, carnivorous animals with fangs such as lions, dogs, wolves, or tigers, predator birds such as falcons, eagles, owls, or vultures, and certain land animals such as snakes [4]. Among these, pig derivatives (components derived from a pig or *Sus scrofa domesticus*) such as pork fat (lard), pork, and porcine gelatines are the most reported components present in food, cosmetics, and pharmaceutical products [5,6]. In addition, some derivatives coming from non-halal animals such as dog meat [7], wild boar meat [8], and rat meat [9] have been reported to be presented in meat-based food products like meatball and sausages; therefore, the identification and confirmation of non-halal components is very urgent.

Halal certification is needed to ensure that products are safe and allowed to be consumed. The halal process is not only related to religious issues but also to fulfill consumer rights. Issues related to halal certification include healthy, organic, environmentally friendly, cruelty-free animal welfare, ethical, and fair-trade aspects, which have made the halal concept popular and highly accepted by all the societies. Halal certification has attracted non-Muslim consumers who come from different ethnic backgrounds, such as Jews (kosher consumers), Americans, Europeans, and Asians, as well as natural and organic consumers, to consume halal products. In addition, in line with awareness among Muslim societies, the demand for halal products has increased tremendously [10]. To meet the demand, a number of public and private organizations have delved into providing halal certification to products [11]. The main purpose of this review is to discuss some molecular spectroscopic techniques for the analysis of non-halal components in food and pharmaceutical products.

## 2. Molecular Spectroscopy

Molecular spectroscopy is the study involving the interaction between electromagnetic radiation in certain frequencies or wavelengths, with analytes at the molecular level. The use of “molecular” terms is intended to the contrary with atomic spectroscopy, in which analytes are analyzed at the atomic level. The different levels of compositions of certain compounds present in non-halal components such as protein, fatty acids, triglycerides, and lipids are main factors which cause the different profile of molecular spectra at specific wavelengths (in ultraviolet-visible spectra and near-infrared spectra) or wavenumbers (mid-infrared). The functional groups responsible for molecular spectra absorption determine the differentiation between halal and non-halal components present in food and pharmaceutical products [12].

Over the past few decades, molecular spectroscopy has gained interest in the analysis of food and pharmaceutical products, including in stages of production and quality control. This method offers several advantages, such as ease in sample preparation, low cost, less time analysis, less solvent (green analytical chemistry), and the ability to monitor several compounds [13]. Molecular spectroscopy has been widely used for the analysis of any products as well as for halal analysis because this method is capable of detecting non-halal substances in samples. The most common types of molecular spectroscopy used for halal analysis are ultraviolet–visible (UV–vis) spectroscopy, vibrational spectroscopy, and nuclear magnetic resonance (NMR) spectroscopy.

Ultraviolet–visible spectroscopy can be used to monitor the interactions of targeted compounds with ultraviolet light (200–400 nm) and visible light (400–800 nm). The molecule in the sample is subjected to UV–vis light; then the electron undergoes excitation from a ground state to an excited state. This excitation is generally known as electronic transitions [14]. UV–vis spectroscopy can be used for both qualitative and quantitative analysis, and the molecules must have chromophores to be analyzed with the UV–vis method. For quantitative analysis, the absorbance based on Lambert-Beer law is used to quantify the non-halal substances. This method has advantages to be applied for non-halal analysis in food and pharmaceutical products because it allows the detection and quantification of the target of analytes/molecules in the presence of the matrix [15].

Vibrational spectroscopy (infrared and Raman) is a non-destructive analytical technique offering fast analysis and cost-effective that makes it an excellent analytical method for food and pharmaceutical product analysis. Infrared spectroscopy either in the mid-infrared region (4000–400 cm^−1^) and near-infrared region (14000–4000 cm^−1^) is the most commonly used method for food analysis [16]. The principle of infrared spectroscopy is based on the interactions between samples with electromagnetic radiation at the infrared region, resulting in vibrational transitions of molecules in the sample. FTIR spectroscopy using the mid-infrared region (4000–400 cm^−1^) has wide application in the analysis of food and pharmaceutical products [17]. FTIR using the ATR (attenuated total reflectance) spectroscopy technique for obtaining spectra of samples offers multiple advantages such as easy in-sample preparation; for instance, the samples can be directly placed on an ATR crystal for measurement, with less time analysis and requiring less solvent. Mid-infrared FTIR spectroscopy enables a wider range analysis of compounds; moreover, it showcases the fingerprint region in the wavenumber region of 1500–900 cm^−1^ that strongly supports the differentiation of non-halal compounds [18]. However, the spectra generated from FTIR measurements are complex, which makes it difficult to be analyzed; therefore, the use of powerful statistical analysis such as chemometrics of multivariate analysis is unavoidable to overcome this problem [19].

Raman spectroscopy is an analytical method based on inelastic scattering, resulting in shifted energy frequencies. Raman scattering relies upon inelastic scattering, whereas elastic scattering results in Rayleigh scattering. In Raman scattering, the molecule is subjected to photons, resulting in excitation from a ground state to the excited state, then undergoing a wavelength shift [20]. Raman and FTIR spectroscopy are complementary methods in some analyses [21]. There are several types of Raman spectroscopy, such as Fourier transform Raman spectroscopy, dispersive Raman spectroscopy, spatially offset Raman spectroscopy, and surface-enhanced Raman spectroscopy [22]. Moreover, Raman spectroscopy offers an imaging technique for fingerprinting and profiling of compounds in samples, which is very useful for sample differentiation. Therefore, the application of Raman spectroscopy for ensuring halal products is obviously promising [23].

Nuclear magnetic resonance (NMR) spectroscopy is a sophisticated molecular spectroscopy that has been extensively used for food and pharmaceutical product analysis, and it has been considered as a potential analytical method for the detection of non-halal substances [24]. The principle of NMR spectroscopy is based on the interaction of molecules with certain radio waves, resulting in the changes of spin direction. NMR provides fingerprint spectra, which makes it useful for sample differentiation, including the detection of non-halal substances [25]. The instruments mostly used for the analysis operate at a frequency of 500–600 MHz. NMR is a versatile molecular spectroscopy technique because of its advantages, such as easy in-sample preparation, less solvent requirements (considered as green analytical chemistry), less time required for analysis, highly reproducible and highly robust, as well as it can be used for the analysis of heterogeneous samples simultaneously [26]. The most common technique used is proton-NMR (^1^H-NMR) spectroscopy because it offers simplicity in sample preparation, fast analysis, and it can be used even for crude extract analysis [27]. Other NMR techniques that play important roles in halal authentication analysis are carbon NMR (^13^C-NMR) and two-dimensional NMR (2D) techniques such as *J*-resolved, HSQC (heteronuclear single quantum correlation), and HMBC (heteronuclear multiple bonds correlation). Research in food analysis has been conducted using NMR spectroscopy, including the authentication of edible oils such as virgin olive oil, cod liver oil, and sesame oil [28]. The benefit of using NMR spectroscopy is the capability for metabolomics analysis, either by metabolite fingerprinting or metabolite profiling, which is very useful for metabolite differentiation in adulteration practices, including the detection of non-halal substance [24]. A combination with chemometrics of multivariate analysis, which can manage the huge dataset generated from NMR measurements, makes it an ideal method for metabolomics analysis of food and pharmaceutical products, as well as halal authentication [29].

## 3. Chemometrics

According to the International Chemometrics Society (ICS), the definition of chemometrics is described as “the science of relating chemical measurements made on a chemical system to the property of interest (such as concentration) through the application of mathematical or statistical methods” [30]. Chemometrics is widely applied in chemical data of molecular spectra and chromatograms. Data from the results of the multi-component analysis, as analyzed using molecular spectroscopy, is complex; therefore, it is very difficult to evaluate. To solve this problem, chemometrics with mathematical and statistical techniques is applied to retrieve more information from the chemical data [31].

In line with the advanced development of statistical software, computer technology, and analytical approaches, the chemometric method has emerged as the leading tool among analytical chemists in order to obtain faster analysis results and shorter product development times [19]. In halal authentication, various chemometric techniques are commonly applied and provide an alternative way to analyze the complex chemical data, namely, chemometric classification analysis and multivariate calibration [6]. Some data pre-processing, such as mean centering, Savitzy–Golay-based derivatization, standard normal variate, baseline corrections, signal correction and compression, spectra normalizations, and multiplicative correction, are also used to treat molecular spectra before being subject to chemometrics analysis.

### 3.1. Chemometrics of Classification

**Figure 1:**
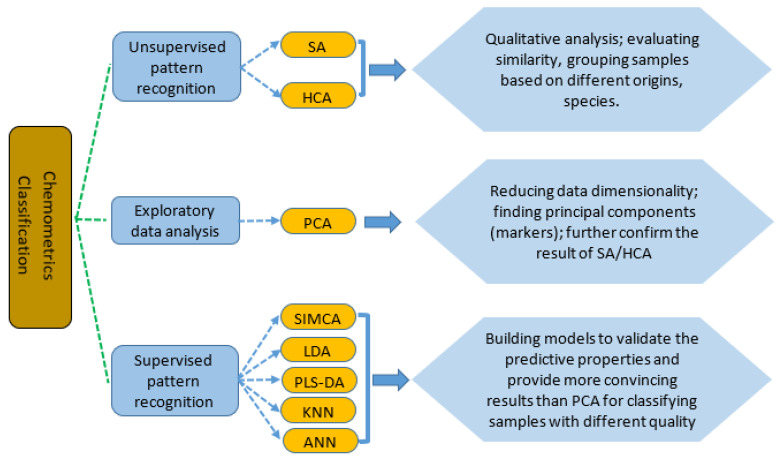
The chemometrics techniques widely applied for the classification of objects. SA = similarity analysis, HCA = hierarchical clustering analysis, PCA = principal component analysis, SIMCA = soft independent modeling of class analogy, LDA = linear discriminant analysis, PLS-DA = partial least squares discriminant analysis, KNN = k-nearest neighbors, and ANN = artificial neural networks [30].

Chemometrics of classification is the most common chemometric technique applied in halal authentication. It is typically performed using three approaches, namely, exploratory data analysis, unsupervised pattern recognition, and supervised pattern recognition techniques, as shown in Figure 1. Exploratory data analysis and unsupervised pattern recognition are commonly used to simplify groups of samples by reducing the amount of original data and gaining better knowledge of chemical data sets. Therefore, the main challenge of these is to remove the redundancy and noise while retaining the meaningful information contained in the original data [32].

Exploration data analysis is a variable (data) reduction technique defining a number of latent variables used to make linear combinations of the original variables, which include principal component analysis (PCA), projection pursuit (PP), and factor analysis (FA). Among these, PCA is the most widely applied compared to PP and FA in the reduction of data dimensionality. Unsupervised pattern recognition differs from exploratory data analysis because the aim of the methods is to detect similarities, whereas exploratory data analysis has no particular preconception as to whether or how many groups will be found. The unsupervised pattern recognition techniques include clustering analysis (CA) and similarity analysis (SA). CA, comprising fuzzy clustering (FC) and hierarchical clustering analysis (HCA), can be used for preliminary evaluation of the information contents in the data matrices. The objects (samples) are classified based on similarities of the used variables [33].

Supervised pattern recognition (SPR) tries to make the class membership of the objects (samples) to a certain group known as training sets. It enables classifying new unknown samples (test samples) in one of the known classes on the basis of variables used. SPR can be differentiated into class-modeling methods and discrimination methods. The most-used class modeling method is known as SIMCA or soft independent modeling of class analogy. SIMCA considers the objects (samples) that fit the class model for a category as part of the class model and classify as non-members those that do not. Discrimination models include linear discriminant analysis (LDA), partial least squares discriminant analysis (PLS-DA), artificial neural networks (ANNs), and k-nearest neighbors (KNNs). All these discrimination models are used to build models using certain variables (for example, FTIR spectra) based on all the categories concerned in the discrimination, whereas disjoint class-modeling methods create a separate model for each category. The most-reported discrimination methods are discriminant analysis, either using linear or partial least square algorithms. LDA is based on linear discriminant functions (LDFs) in which the variance ratio of between-class membership of objects is minimized, while the variance ratio of within-class objects is maximized. PLS-DA is intended to find the variables and directions in the multivariate space capable of discriminating the established classes in the calibration set [34]. ANNs consider finding the most appropriate grouping of training, learning, and transfer function for classifying the data sets (variables) with a growing number of features and classified sets. In a simple form, ANNs try to imitate the operation of neurons in the brain [35]. KNN is one of the most popular classification techniques based on distance algorithms. KNN is based on measuring the distances between the training samples and test samples to determine the final classification output [36].

### 3.2. Chemometrics of Quantification

For quantitative analysis of non-halal components in food and pharmaceutical products, the chemometrics of multivariate calibration is usually used to predict the levels of analyte(s) of interest in unknown samples [37]. Calibration is the mathematical relationship between the predictor (independent variables) and response variables (dependent variables). The chemometrics of multivariate calibration uses several variables, such as employing the absorbance values at several wavelengths or wavenumbers region. Multivariate calibration is commonly used to develop calibration and validation models capable of correlating the actual values of analytes as determined by the reference method and predicted values using several variables assessed [38].

Various multivariate calibrations have been used for quantitative analysis of non-halal components, including stepwise multiple linear regression (SMLR), principal component regression (PCR), and partial least square regression (PLSR) [39]. These calibrations are considered as inverse calibration in which concentrations (in the *y*-axis) are modeled using absorbances at several wavelengths/wavenumbers (*x*-axis) [40]. The accuracy of calibration and validation models using multivariate calibration was evaluated by the coefficient of determination (R^2^) for the relationship between two variables, while the precision of models was assessed by the root mean square error of calibration (*RMSEC*) and root mean square error of prediction (*RMSEP*). *RMSEC* and *RMSEP* were obtained using these equations:

(1) $$RMSEC=\sqrt{\frac{\sum_{i=1}^{m}{\left(\hat{Y}i-Yi\right)}^{2}}{M-1}}$$

(2) $$RMSEP=\sqrt{\frac{\sum_{i=1}^{n}{\left(\hat{Y}i-Yi\right)}^{2}}{N}}$$

**Figure 2:**
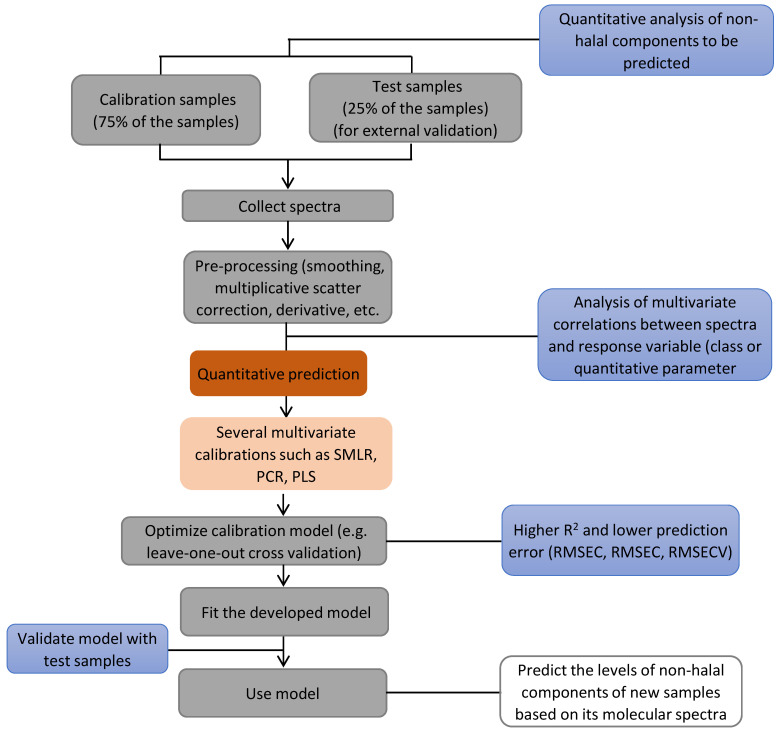
Scheme of quantitative analysis of non-halal components in food and pharmaceutical products assisted by multivariate calibration [42].

*Yi* and *Ŷi* represent the actual and predicted value of analytes, while *M* and *N* are the numbers of data in the calibration and validation sets [41]. Figure 2 explains the role of multivariate calibration for quantifying non-halal components in the evaluated samples.

The main advantage of multivariate calibration is the reliability of prediction results for unknown samples obtained. However, multivariate calibrations have the main disadvantage, namely, the over-fitting of the model. Overfitting is the over-optimistic performance of multivariate calibrations in calibration datasets, but the performance in validation datasets is not acceptable; as a consequence, cross-validation of the leave-one-out technique can be used to assess this problem. In cross-validation, one of the calibration samples is left out from multivariate calibration models used, and the remaining calibration samples are exploited for developing a new calibration model. The removed sample is then calculated using the newly developed PLS model. This procedure was repeated by leaving one by one of the calibration samples. The statistical parameters used to evaluate the performance of cross-validation is R^2^ (for accuracy of the model), as well as the root mean square error of cross-validation (RMSECV) and the predicted residual error sum of squares or PRESS (for the precision of the model) [35].

## 4. Application of Molecular Spectroscopy Combined with Chemometrics for Analysis of Non-Halal Components

Molecular spectroscopy (UV–vis, infrared, Raman, and NMR) combined with chemometrics techniques have been applied for the analysis of non-halal components, especially pig derivatives and non-halal meats in food and pharmaceutical products.

### 4.1. UV–vis Spectroscopy

Ultraviolet–visible spectroscopy has been used for the differentiation of fish, porcine, and bovine gelatines based on measurement of the degree of browning during Maillard reaction of different types of reducing sugars. Among reducing sugars evaluated, D-(+)-xylose has the highest value of browning value, as indicated by the highest absorbance values at 420 nm compared to other sugars. The degrees of browning products (Maillard reaction products) combined with chemometrics of PCA can differentiate the sources of gelatines. According to the loading plot in PC1 and PC2, D-(+)-xylose is the most contributing variable in classification among gelatines [43]. Previously, some parameters affecting the Maillard reaction were investigated using response surface methodology [44]. The effects of initial pH, temperature, and heating time toward the browning intensity of melanoidin have been evaluated. The increase of initial pH, temperature, and heating time is associated with an enhanced browning intensity of Maillard reaction products.

Another study involved the evaluation of several factors contributing to the Maillard reaction, resulting in the browning index measured using visible spectroscopy at 420 nm for differentiation of gelatine sources being investigated [45]. The parameters evaluated included temperature, time, and the presence of metal ion Cu^2+^. The optimal reaction was obtained using the temperature of the water bath at 95 °C for 9 h with a concentration of metal ion Cu^2+^ of 5 m. This optimum condition influences the differentiation of bovine gelatine compared to fish and porcine gelatine. Maillard reaction, as analyzed using UV–vis spectroscopy, is one of the convenient protocols for authentication of halal gelatine.

### 4.2. Infrared Spectroscopy

Among molecular spectroscopies, Fourier transform infrared spectroscopy is the most reported method used for the analysis of non-halal components in any products. Due to its advantages as fingerprint analytical techniques, FTIR spectroscopy, in combination with chemometrics, is widely applied for the rapid identification of non-halal components, including pig derivatives and several types of non-halal meats such as wild boar meat, dog meat, and rat meat [46]. Che Man et al. [47] have used FTIR spectroscopy combined with chemometrics of principal component analysis (PCA) and cluster analysis (CA) for the identification and confirmation of lard and other edible fats and oils. Lard, obtained from rendering adipose tissues of pigs, and other fats and oils were subjected to FTIR spectra measurement using the sampling technique of attenuated total reflectance (ATR) in normal spectra at wavenumbers (1/λ) of 6000–650 cm^−1^. Using PCA, lard and other contaminants could be separated along with the first principal component (PC1) and second principal component (PC2), accounting for variations of 44.1% and 30.2%, respectively, using absorbance values picked at 16 different wavenumbers as variables. Based on the loading plot, the variables most contributing to the separation on PC1 and PC2 were absorbance values at 2853, 2922, and 1465 cm^−1^. In addition, CA could make clustering of lard and other fats and oils, including chicken fat, cod liver oil, corn oil, rice bran oil, soybean oil, sunflower oil, sesame oil, extra virgin olive oil, pumpkin seed, walnut oil, palm oil, and canola oil using the same variables used in PCA analysis. Lard appeared at the separate clusters from other fats and oils observed using a CA dendrogram.

**Figure 3:**
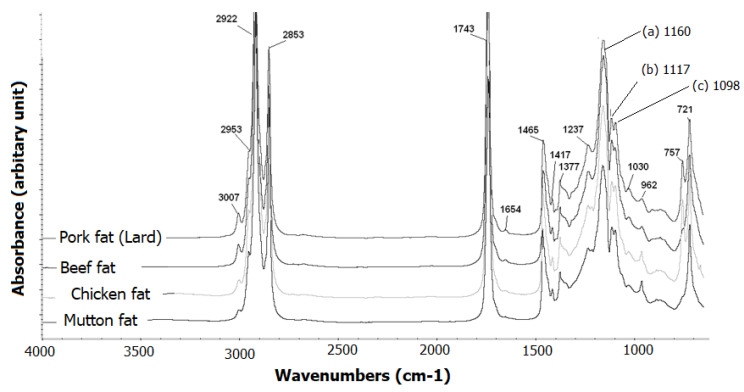
FTIR spectra of lard, beef fat, chicken fat, and mutton fat at wavenumbers of 4000–650 cm^−1^. Taken with permission from the PhD thesis of Abdul Rohman [49].

For quantitative analysis, FTIR spectroscopy combined with chemometrics of partial least square regression (PLSR) has been successfully used for the simultaneous analysis of lard in the quaternary mixtures with chicken fat, mutton fat, and beef fat [48]. Figure 3 shows the FTIR normal/original spectra of these animal fats. These FTIR spectra look similar and revealed typical characteristic of absorption bands of triacylglycerols. The peak at 3007 cm^−1^ corresponds to the stretching of vinylic C-H. The stretching vibrations of methylene (-CH_2_-) and methyl (-CH_3_) groups can be seen at frequencies of 2922 and 2853 cm^−1^, respectively. Methylene and methyl groups are also observed at 1465 and 1377 cm^−1^ due to their bending vibrations. The carbonyl (C=O) absorption of ester linkage is observed at 1743 cm^−1^ with strong intensity. The bands at 1237, 1160, 1117, 1098, and 721 cm^−1^ are the results from the overlapping of the CH_2_-rocking vibrations and the out-of-plane bending vibration of *cis*-disubstituted olefins. However, using detailed investigation, the peak intensities of lard could be differentiated from others, especially in peaks 1160 (a), 1117 (b), and 1098 cm^−1^ (c).

Several FTIR spectral regions and their combinations have been optimized for developing a PLS calibration model intended for the quantification of lard. The selection of wavenumbers regions was based on the highest values of R^2^ and the lowest values of RMSEC and RMSEP. Finally, first derivative spectra at selected fingerprint regions of 1500–1000 cm^−1^ were suitable for the quantitative analysis of lard in a quaternary mixture. The values of R^2^ and RMSEC obtained were 0.9997 and 0.773%, respectively.

In food products, FTIR spectroscopy combined with chemometrics of PLSR could be used for quantitative analysis of non-halal meat of pork in beef meatballs for adulteration issues. PLSR at a selected fingerprint wavenumber region (1200–1000 cm^−1^) was capable of quantifying lard, a lipid fraction extracted from meatballs containing pork, with R^2^ and RMSEC values of 0.996% and 0.712%, respectively [50]. In addition, discriminant analysis (DA) at the same wavenumbers was also successful in discriminating between beef meatballs and pork meatballs. Pork in beef meatballs also can be analyzed using the lipid fractions extracted from meatball broth. The chemometrics of PCA could classify meatball broth with and without pork in the analyzed calibration samples using a variable of absorbance values at 1200–1000 cm^−1^. Meanwhile, lard extracted from pork meatballs was quantified by PLSR using absorbances at wavenumbers of 1018–1284 cm^−1^, providing R^2^ and RMSEC values of 0.9975% and 1.34% (*v*/*v*), respectively. The results indicated that FTIR spectroscopy, in combination with PCA and PLS, is a rapid and reliable technique for detection and quantification of pork in meatballs for halal authentication studies [51]. Table 1 compiled the application of FTIR spectroscopy combined with multivariate analyses (chemometrics) used for the analysis of non-halal components in food and pharmaceutical products.

### 4.3. Raman Spectroscopy

Raman spectroscopy has been used for differentiation and quantification of lard in mixtures with other animal fats and oils such as beef tallow, chicken fat, and duck oil. The analysis was performed using a 785-nm laser diode. The spectra were recorded in the wavenumber region of 1800–700 cm^−1^ using a resolution of 1.25 cm^−1^. Chemometrics of principal component analysis (PCA) was successfully used for the classification of these four types of animal fats. The combination of Raman and chemometrics of partial least square (PLS) was also successfully applied for the quantification of lard in binary mixtures with duck oil and beef tallow [69]. The model showed high correlation coefficient values for lard in beef tallow (0.96674) and lard in duck oil (0.97148), indicating the good fit of the model [70].

Fatty acid analysis of pork backfat has been performed using Raman spectroscopy and chemometrics. Samples were subjected to Raman spectroscopy using a 785 nm laser diode, and the spectra were recorded in the wavenumber region of 1800–200 cm^−1^. PCA was successfully applied to differentiate between inner and outer fat layers from pork [71]. PCA score plot showed that inner and outer fat layers were well separated using PCA, indicating that they were systematically different. Investigation on PCA loading score resulting that peaks at the wavenumber of 1296, 1128, and 1061 cm^−1^ have positive contributions in sample differentiation. Chemometrics of PLS has also been used for the prediction of fatty acids, either individual or total fatty acids, using Raman spectroscopy and gas chromatography for reference to fatty acid concentration. All models showed high values of R^2^ and lower RMSEC values that confirmed the accuracy and precision of the PLS model [72].

Another study of L-cysteine detection in wheat flour has been successfully performed using Raman micro-spectroscopy coupled with chemometrics of HCA (hierarchical cluster analysis) and PCA. Raman micro-spectroscopy is one of Raman techniques equipped with an optical microscope that enables a non-invasive acquisition spectrum with resolution down to 1 µm. L-cysteine is widely used as food additives; however, the use of L-cysteine in wheat flour is forbidden in Turkey, considering its source. L-cysteine is commonly obtained from animals such as pigs, cows, and ducks. Moreover, it can also be obtained from humans, such as human hair [73]. Therefore, it is forbidden to be mixed with food products related to the halal status of the products. The combination of Raman spectroscopy measured using a 532 nm diode laser in the wavenumber region of 2100–400 cm^−1^ and chemometrics of PCA using first derivative spectra was successfully used to differentiate authentic wheat flour and wheat flour adulterated with L-cysteine. Moreover, analysis using HCA resulted in well-separated clusters between adulterated and unadulterated samples, with L-cysteine observed from the dendrogram [74]. It could be suggested that Raman micro-spectroscopy, in combination with chemometrics, provides an adequate method for the detection of L-cysteine adulteration in wheat flour.

### 4.4. Nuclear Magnetic Resonance (NMR) Spectroscopy

Nuclear magnetic resonance (NMR) spectroscopy emerges as a sophisticated method for food and pharmaceutical product analysis [75]. The use of NMR spectroscopy for halal authentication in food products has been developed recently. ^1^H-NMR spectroscopy has been successfully used for the detection and quantification of lard in binary mixtures with butter. Butter is one of the milk-derived products which has many functions and benefits in food and dairy products. The combination of ^1^H-NMR spectroscopy with chemometrics of partial least square (PLS) was successfully used for authentication of butter from lard. The PLS model was linear and showed a strong correlation between the actual value and predicted/calculated values of lard presented by its high R^2^ value (more than 0.998) for both calibration and validation models. The lower RMSEC (0.0091) and RMSEP (0.0090) values were obtained, indicating lower errors and high precision in the PLS model [76].

Carbon NMR (^13^C-NMR) spectroscopy has also been successfully used for authentication of butter adulterated lard instead of the ^1^H-NMR spectroscopy technique. The spectra generated from ^13^C-NMR measurements are more complex, but they are more informative because they contain many important signals of interest compounds [77]. The resonance of some compounds, such as fatty acids and triacyclglycerols, could be used for sample differentiation from several sources. For instance, the region of palmitic acid and olefinic could be used to distinguish lard and butter. Moreover, two-dimensional (2D) NMR spectroscopy also possesses advantages for the authentication of fats and oils. Heteronuclear multiple bond correlation (HMBC) is the sophisticated 2D NMR technique in NMR spectroscopy, which shows more detailed information about nuclei correlation either carbon to carbon or carbon to proton. The study on butter adulterated lard using the HMBC technique found the signals of palmitoyloleoyllinoleoyl (POL) [78].

NMR spectroscopy has been employed for the authentication of milk fat blended with animal and vegetable fats. Milk fat has the highest price among fats, so that it has the potential to be adulterated with other fats [79]. ^13^C-NMR spectroscopy was successfully used for authentication of milk fat blended with pork (lard) and margarine. The compound of butyrate was found as a marker to ensure the authenticity of milk fat. Butyrate is specific for milk fat compound, and no butyrate signals were observed either in lard or in margarine. The butyryl backbone appeared in the chemical shift of 173.13, 35.94, 18.37, and 13.63 ppm. Quantification of butyrate was also successfully performed using NMR, and the result was compared to the gas chromatography method. These methods showed high regression coefficient (R^2^ more than 0.999) for quantification of butyrate indicating that NMR could be used for quantification of butyrate for detection of adulteration in milk fat from lard and margarine [80].

^1^H-NMR spectroscopy, in combination with chemometrics of multivariate analysis, has been successfully applied for authentication of heparin, a pharmaceutical product derived from animal tissue that plays an important role in blood coagulation [81]. Some animal species could be the sources for heparin, including pigs. ^1^H-NMR spectroscopy, coupled with principal component analysis (PCA), was successfully used to differentiate bovine, ovine, and porcine heparin. Heparin samples were extracted using deuterium oxide (D_2_O), then measured using a 600 MHz NMR spectrometer. Another chemometrics technique, namely K-means, was also successfully used to classify bovine, ovine, as well as porcine, heparin. K-means is an unsupervised pattern recognition technique which is used for group clustering using pre-defined K variables. The less variation within a cluster, the more homogenous variables within the same cluster. Bovine, ovine, and porcine heparin samples were completely separated using both PCA and K-means. It suggested that ^1^H-NMR spectroscopy, in combination with chemometrics, offers a powerful technique for the authentication of pharmaceutical products such as heparin [82].

## 5. Methods

During performing this review, we explored some databases such as the Science Citation Index, PubMed, Medline, Scopus, and Google Scholar to identify and to download the abstracts, reports, review articles, and research papers related to the molecular spectroscopic techniques for analysis of non-halal components. The keywords used during searching of information were molecular spectroscopy (or UV–vis spectroscopy, or infrared spectroscopy, or Raman spectroscopy or NMR spectroscopy) + pig derivatives (or non-halal meat, or pork, or rat meat, or dog meat, or wild boar meat) + halal authentication.

## 6. Conclusions

Molecular spectroscopy, based on the interaction of electromagnetic radiation (EMR) with non-halal components in molecular levels, including ultraviolet-visible, infrared, Raman, and nuclear magnetic resonance (NMR) spectroscopies, revealed promising tools for screening and identification of pig derivatives and non-halal meats in food and pharmaceutical products. Combined with chemometrics of classification and quantification, molecular spectroscopy has emerged as rapid and reliable analytical techniques for analysis of pig derivatives (lard, pork, porcine gelatin) and non-halal meats (rat meat, wild boar meat, and dog meat) with acceptable analytical performance in terms of accuracy and precision. However, the molecular spectroscopy methods need to be confirmed using other instruments such as gas chromatography–mass spectrometry (GC–MS), two-dimensional gas chromatography–mass spectrometry (GC × GC–MS), liquid chromatography–tandem mass spectrometry (LC–MS/MS), and real-time polymerase chain reaction (RT-PCR) methods.

## Figures and Tables

**Table 1:** The application of near-infrared (NIR) and mid-infrared (MIR) spectroscopy with several chemometrics techniques for analysis of non-halal components in food and pharmaceutical products.

Non-Halal Components	Issue	Infrared (NIR/MIR) Spectroscopy Condition	Chemometrics Techniques	Results	References
Lard (pork fat)	Adulteration of lard in palm oil	NIR at wavelength 950–1650 nm using transflectance and transmission sampling techniques	Classification using SIMCA, quantification using PLS	SIMCA can classify palm oil and palm oil adulterated with lard with model accuracy of 0.93 (transflectance) and 0.95 (transmission). NIR can predict lard content described by an equation relating between actual value of lard (x) and NIR-PLS predicted value (y) as: Y = 0.9987x + 0.02032 (transflectance)Y = 0.9994x + 0.01024 (transmission)	[52]
Lard	Adulteration of beef with pork through analysis of lard	The extraction was performed using Soxhlet apparatus at 70 °C for 6 h with n-hexane. FTIR normal spectra at 1/λ 1200–1000 cm^−1^ using ATR technique	Classification using PCA and quantification with PLS regression	FTIR spectra combined with PCA could classify sausages with pork and beef. PLS gave an equation of predicted value = 0.921 x (actual value) + 4.623 R^2^ = 0.985 and RMSEC = 2.094%; RMSEP = 4.77% RMSECV = 5.12%.	[53]
Lard	Analysis of lard in crackers “rambak” (Foods consumed among Indonesian people made from various kinds of animal skin)	The “rambak” crackers containing pigskin and cow skin was subjected to Soxhlet extraction using hexane. ATR-FTIR spectra at 1/λ 1200–1000 cm^−1^	PLS regression	PLS regression could predict lard extracted from “rambak” crackers with *R*^2^ (calibration) of 0.946 with RMSEC of 2.77%. For validation models, *R*^2^ of 0.997 and RMSEP of 2.77.	[54]
Lard	Analysis of lard in “rambak” crackers containing buffalo skin	The “rambak” crackers was extracted using Soxhlet procedure with hexane as extracting solvent. ATR-normal FTIR spectra at 1/λ 1200–1000 cm^−1^	Classification using PCA and quantification with PLS regression	PCA could classify “rambak” crackers according to animal skin (pigskin and buffalo skin). PLS regression could predict pigskin in rambak with R^2^ of 0.96, RMSEC of 2.56%, and RMSEP of 1.10%.	[55]
Lard	Analysis of lard in bread formulation	Lard in bread was extracted using Bligh and Dyer method by extensive vortexing at each step. Second derivative spectra at 1/λ 1190–900 cm^−1^ using ATR technique	PLS regression for quantification	PLS using the selected FTIR spectra region could quantify lard in bread successfully with detection limit of 1% *w*t/*w*t	[56]
Lard (pork fat)	Adulteration of chicken fat with pork fat in food products	Normal spectra at 1/λ 1236 and 3007 cm^−1^ using ATR technique	Classification using PCA	Combination of FTIR spectra and chemometrics could classify lard in chicken fat, pure lard, food containing lard, palm oil, and chicken fat	[57]
Lard	Differentiation of lard chicken, mutton, tallow- and palm-based shortening	Samples are heated at different temperatures (120, 180 and 240 °C) and time (30, 60, 120 and 180 min) and normal FTIR spectra at 4000–650 cm^−1^ were evaluated for differentiation	Classification using PCA, *k*-mean cluster analysis and LDA	The combination of PCA with *k*-mean CA was capable of differentiating the heated lard and other samples according to its origin. LDA method was successfully used to classify 80.5 % of samples according to its group	[58]
Lard	Analysis of lard in crude palm oil (CPO) for authenticity issue	Lard in the mixture with CPO using ATR at the combined wavenumbers of 1481–999 and 1793–1650 cm^−1^	PLS regression	PLS could predict the levels of lard in CPO with R^2^ value of 0.998 and RMSEC of 1.291% (*v*/*v*) and RMSECV value of 0.838% (*v*/*v*).	[59]
Lard	Analysis of lard in palm oil	The samples were directly subjected to short wave near-infrared spectroscopy (NIR) at wavelength 800–1600 nm and measured with transflectance and transmission modes	Spectra were subjected to variable selection. Classification using SIMCA, quantification with PLS	SIMCA algorithm could classify lard and palm oil mixed with lard with accuracy level of >0.95 for both transflectance and transmission modes. PLS regression could predict the levels of lard in palm oil with R^2^ of 0.9987 (transflectance) and 0.9994 (transmission) with RMSEC of 0.5931 (transflectance) and 0.6703 (transmission).	[52]
Lard	Detection of the presence of lard in pure ghee (heat clarified milk fat)	Normal FTIR spectra at combined 1/λ region of 3030–2785, 1786–1680, 1490–919 cm^−1^	Classification using SIMCA, quantification using PLS	Pure ghee and the one adulterated with lard could be classified using PCA. Using SIMCA, 90% of the samples were classified into their respective class. PLSR could quantify lard with R2 >0.99 in calibration and prediction models. Detection limit reported was 3% *w*t/*w*t	[60]
Lard	Analysis of lard in cheese samples	FTIR normal spectra at wavenumbers of 700, 1140–1070, 756 and 720 cm^−1^	Quantification of lard using PLS regression	PLS could quantify the level of lard in cheese samples successfully	[61]
Lard	Analysis of lard in lipstick	Lard was extracted from lipstick using saponification method followed by liquid/liquid extraction with hexane/dichlorometane (DCM)/ethanol/water, saponification method followed by liquid/liquid extraction with DCM/ethanol/water, and Bligh and Dyer method. ATR-FTIR spectra were measured at 1200–800 cm^−1^	Classification of lipstick with and without lard was performed using PCA, while quantification of lard was performed using PLS regression	PCA could classify lipstick with lard and without lard in its formulation with PC1 accounted for 63.7%, and PC2 accounted for 26.4% (90.1% of the variance is described by PC1 and PC2). PLS is capable of predicting the amount of lard in lipstick formulation with the equation y (predicted value) = 1.0070 x (actual value) − 4563 (R^2^ = 0.9956) in calibration model and y = 0.9811x + 0.3381 (R^2^ of 0.9970) in validation equation.	[62]
Lard, lard olein (LO) and lard stearin (LS)	Differentiation of LO and LS from other common animal fats	Normal spectra at wavenumbers region (4000–650 cm^−1^)	PCA	Due to its fingerprint nature, FTIR spectra combined with PCA could differentiate and could classify LO and LS from chicken fat, lard, beef fat, and mutton fat	[63]
Pork	Adulteration of beef meatball with pork	The extraction of lard is performed using concentrated hydrochloric acid as a hydrolytic agent and petroleum benzene as solvent extraction. Normal FTIR spectra 1000–1200 cm^−1^ using ATR technique	Classification using PCA and quantification using PLS regression	PLS regression offered good relationship between actual value and predicted value of lard with FTIR predicted value with R^2^ 0.997 and standard error of calibration of 0.04%. PCA could classify beef meatball and beef meatball mixed with pork	[64]
Dog meat	Adulteration of dog meat in beef meatball	The lipid fraction of meatball was obtained using Bligh-Dyer and Folch extraction methods. ATR-Normal FTIR spectra at 1700–700 cm^−1^.	Classification using PCA and quantification using PLS regression	FTIR spectroscopy, coupled with chemometrics at 1700–700 cm^−1^, is capable of classifying dog meatballs and beef meatballs. PLS offered reliable quantitative analysis of dog meat in beef meatballs with acceptable statistical results	[65,66]
Porcine gelatin	Analysis of porcine gelatin in candies and its classification from other gelatin types	Direct analysis using ATR technique. FTIR spectra were analyzed at 1734–1528 cm^−1^	Classification between halal gelatin and non-halal gelatin using HCA, PCA, and PLS-DA	Gummy candy samples could be classified accurately according to its sources with accuracy levels of 100% using Ward’s algorithm (HCA), PLS-DA, and PCA. The results were confirmed by real-time polymerase chain reaction	[67]
Porcine gelatin	Differentiation between porcine gelatin and bovine gelatin	Direct analysis using ATR at combined region of 3290–3280 and 1660–1200 cm^−1^	PCA and DA	DA based on the Cooman’s plot obtained using the software TQ Analyst could classify and discriminate gelatines without any misclassification exploiting the same peaks used in PCA analysis	[68]

RMSEC = root mean square error of calibration; RMSEP = root mean square error of prediction; RMSECV = root mean square error cross-validation; PCA = principal component analysis; DA = discriminant analysis; LDA = linear discriminant analysis; SIMCA = soft independent modeling class analogy; HCA = hierarchical cluster analysis; PLS = partial least square; PLS-DA = partial least square discriminant analysis; SIMCA = soft independent modeling class of analogy.

## References

[1] Ahmad A.N., Ungku Zainal Abidin U.F., Othman M., Abdul Rahman R. (2018). Overview of the halal food control system in Malaysia. Food Control.

[2] Lubis H.N., Mohd-Naim N.F., Alizul N.N., Ahmed M.U. (2016). From market to food plate: Current trusted technology and innovations in halal food analysis. Trends Food Sci. Technol..

[3] Jamaludin M.A., Rahman N.D.A., Fadzillah N.A., Ramli M.A. (2018). Preparation and Processing of Religious and Cultural Foods.

[4] Mursyidi A. (2013). The Role of chemical analysis in the halal authentication of food and pharmaceutical products. J. Food Pharm.Sci..

[5] Erwanto Y., Rohman A., Arsyanti L., Pranoto Y. (2018). Identification of pig DNA in food products using polymerase chain reaction (PCR) for halal authentication—A review. Int. Food Res. J..

[6] Rohman A., Salamah N. (2018). The employment of spectroscopic techniques coupled with chemometrics for authentication analysis of halal pharmaceuticals. J. Appl. Pharm. Sci..

[7] Rahman M.M., Ali M.E., Hamid S.B.A., Mustafa S., Hashim U., Hanapi U.K. (2014). Polymerase chain reaction assay targeting cytochrome b gene for the detection of dog meat adulteration in meatball formulation. Meat Sci..

[8] Guntarti A., Martono S., Yuswanto A., Rohman A. (2017). Analysis of beef meatball adulteration with wild boar meat using real-time polymerase chain reaction. Int. Food Res. J..

[9] Widyasari Y.I., Sudjadi, Rohman A. (2015). Detection of rat meat adulteration in meat ball formulations employing real time PCR. Asian J. Anim. Sci..

[10] Latif I.A., Mohamed Z., Sharifuddin J., Abdullah A.M., Ismail M.M. (2014). A Comparative analysis of global halal certification requirements. J. Food Prod. Mark..

[11] van der Spiegel M., van der Fels-Klerx H.J., Sterrenburg P., van Ruth S.M., Scholtens-Toma I.M.J., Kok E.J. (2012). Halal assurance in food supply chains: Verification of halal certificates using audits and laboratory analysis. Trends Food Sci. Technol..

[12] Kumar Y., Chandrakant Karne S. (2017). Spectral analysis: A rapid tool for species detection in meat products. Trends Food Sci. Technol..

[13] Cozzolino D. (2014). An overview of the use of infrared spectroscopy and chemometrics in authenticity and traceability of cereals. Food Res. Int..

[14] Pavia D.L., Lampman G.M., Kriz G.S. (2001). Introduction to Spectroscopy.

[15] Behera S. (2012). UV-Visible spectrophotometric method development and validation of assay of paracetamol tablet formulation. J. Anal. Bioanal. Tech..

[16] Qi L.M., Zhang J., Liu H.G., Li T., Wang Y.Z. (2017). Fourier transform mid-infrared spectroscopy and chemometrics to identify and discriminate *Boletus edulis* and *Boletus tomentipes* mushrooms. Int. J. Food Prop..

[17] Rohman A. (2017). The use of infrared spectroscopy in combination with chemometrics for quality control and authentication of edible fats and oils: A review. Appl. Spectrosc. Rev..

[18] Moros J., Garrigues S., Guardia M. (2010). Vibrational spectroscopy provides a green tool for multi-component analysis. TrAC Trends Anal. Chem..

[19] Worley B., Powers R. (2013). Multivariate analysis in metabolomics. Curr. Metabolomics..

[20] Craig A.P., Franca A.S., Irudayaraj J. (2013). Surface-enhanced Raman spectroscopy applied to food safety. Annu. Rev. Food Sci. Technol..

[21] Kalantri P.P., Somani R.R., Makhija D.T. (2010). Raman spectroscopy: A potential technique in analysis of pharmaceuticals. Der Chem. Sin..

[22] Chen D.D., Xie X.F., Ao H., Liu J.L., Peng C. (2017). Raman spectroscopy in quality control of Chinese herbal medicine. J. Chinese Med. Assoc..

[23] Georgouli K., Martinez Del Rincon J., Koidis A. (2017). Continuous statistical modelling for rapid detection of adulteration of extra virgin olive oil using mid infrared and Raman spectroscopic data. Food Chem..

[24] Dais P., Hatzakis E. (2013). Quality assessment and authentication of virgin olive oil by NMR spectroscopy: A critical review. Anal. Chim. Acta.

[25] Petrakis E.A., Cagliani L.R., Polissiou M.G., Consonni R. (2015). Evaluation of saffron (*Crocus sativus* L.) adulteration with plant adulterants by1H NMR metabolite fingerprinting. Food Chem..

[26] Windarsih A., Rohman A., Swasono R.T. (2019). Application of ^1^H-NMR based metabolite fingerprinting and chemometrics for authentication of *Curcuma longa* adulterated with *C. heyneana*. J. Appl. Res. Med. Aromat. Plants.

[27] Awin T., Mediani A., Shaari K., Faudzi S.M.M., Sukari M.A.H., Lajis N.H., Abas F. (2016). Phytochemical profiles and biological activities of *Curcuma* species subjected to different drying methods and solvent systems: NMR-based metabolomics approach. Ind. Crops Prod..

[28] Kim H.K., Choi Y.H., Verpoorte R. (2010). NMR-based metabolomic analysis of plants. Nat. Protoc..

[29] Danezis G.P., Tsagkaris A.S., Camin F., Brusic V., Georgiou C.A. (2016). Food authentication: Techniques, trends & emerging approaches. TrAC—Trends Anal. Chem..

[30] Kundu M., Kundu P.K., Damarla S.K. (2018). Chemometric Monitoring: Product Quality Assessment, Process Fault Detection and Applications.

[31] Huang Y., Wu Z., Su R., Ruan G., Du F., Li G. (2015). Current application of chemometrics in traditional Chinese herbal medicine research. J. Chromatogr. B Anal. Technol. Biomed. Life Sci..

[32] Berrueta L.A., Alonso-Salces R.M., Héberger K. (2007). Supervised pattern recognition in food analysis. J. Chromatogr. A.

[33] Møller S.F., von Frese J., Bro R. (2005). Robust methods for multivariate data analysis. J. Chemom..

[34] Gad H.A., El-Ahmady S.H., Abou-Shoer M.I., Al-Azizi M.M. (2013). Application of chemometrics in authentication of herbal medicines: A review. Phytochem. Anal..

[35] Miller J.N., Miller J.C. (2010). Statistics and Chemometrics for Analytical Chemistry.

[36] Ali N., Neagu D., Trundle P. (2019). Evaluation of k-nearest neighbour classifier performance for heterogeneous data sets. SN Appl. Sci..

[37] Biancolillo A., Marini F. (2018). Chemometric methods for spectroscopy-based pharmaceutical analysis. Front. Chem..

[38] Rohman A., Putri A.R. (2019). The chemometrics techniques in combination with instrumental analytical methods applied in Halal authentication analysis. Indones. J. Chem..

[39] Bro R. (2003). Multivariate calibration: What is in chemometrics for the analytical chemist?. Anal. Chim. Acta.

[40] Rohman A., Silawati D., Sudjadi, Riyanto S. (2015). Simultaneous determination of sulfamethoxazole and trimethoprim using UV spectroscopy in combination with multivariate calibration. J. Med. Sci..

[41] Pebriana R.B., Rohman A., Lukitaningsih E. (2017). Sudjadi Development of FTIR spectroscopy in combination with chemometrics for analysis of rat meat in beef sausage employing three lipid extraction systems. Int. J. Food Prop..

[42] Nunes C.A. (2014). Vibrational spectroscopy and chemometrics to assess authenticity, adulteration and intrinsic quality parameters of edible oils and fats. Food Res. Int..

[43] Hamid A.H., Nurrulhidayah A.F., Sani M.S.A., Muhammad N.W.F., Othman R., Rohman A. (2020). Discrimination of porcine and bovine gelatines based on reducing sugar types on maillard reaction. Food Res..

[44] Ismarti I., Triyana K., Fadzilah N.A., Salleh H.M., Nordin N.F.H. (2020). Optimisation of the Maillard reaction of bovine gelatine-xylose model using response surface methodology. Food Res..

[45] Hamid A.H., Elgharbawy A.A., Rohman A., Rashidi O., Hammed H., Nurrulhidayah A.F. (2019). Optimisation of browning index of maillard reaction in gelatine powder by response surface methodology (RSM) for halal authentication. Food Res..

[46] Rohman A. (2019). The employment of Fourier transform infrared spectroscopy coupled with chemometrics techniques for traceability and authentication of meat and meat products. J. Adv. Vet. Anim. Res..

[47] Che Man Y.B., Rohman A., Mansor T.S.T. (2011). Differentiation of lard from other edible fats and oils by means of Fourier transform infrared spectroscopy and chemometrics. JAOCS J. Am. Oil Chem. Soc..

[48] Rohman A., Che Man Y.B. (2011). The optimization of FTIR spectroscopy combined with partial least square for analysis of animal fats in quartenary mixtures. Spectroscopy.

[49] Rohman A. (2009). Application of fourier transform infrared spectroscopy for analysis, authentication and monitoring of oxidative stability of edible oils. Ph.D. Thesis.

[50] Rohman A., Sismindari, Erwanto Y., Che Man Y.B. (2011). Analysis of pork adulteration in beef meatball using Fourier transform infrared (FTIR) spectroscopy. Meat Sci..

[51] Kurniawati E., Rohman A., Triyana K. (2014). Analysis of lard in meatball broth using Fourier transform infrared spectroscopy and chemometrics. Meat Sci..

[52] Basri K.N., Hussain M.N., Bakar J., Sharif Z., Khir M.F.A., Zoolfakar A.S. (2017). Classification and quantification of palm oil adulteration via portable NIR spectroscopy. Spectrochim. Acta—Part A Mol. Biomol. Spectrosc..

[53] Guntarti A., Ahda M., Kusbandari A., Prihandoko S. (2019). Analysis of lard in sausage using Fourier transform infrared spectrophotometer combined with chemometrics. J. Pharm. Bioallied Sci..

[54] Erwanto Y., Muttaqien A.T., Sugiyono, Sismindari, Rohman A. (2016). Use of Fourier transform infrared (ftir) spectroscopy and chemometrics for analysis of lard adulteration in “rambak” crackers. Int. J. Food Prop..

[55] Muttaqien A.T., Erwanto Y., Rohman A. (2016). Determination of buffalo and pig “rambak” crackers using FTIR spectroscopy and chemometrics. Asian J. Anim. Sci..

[56] De Cicco M., Siano F., Iacomino G., Iannaccone N., Di Stasio L., Mamone G., Volpe M.G., Ferranti P., Addeo F., Picariello G. (2019). Multianalytical detection of pig-derived ingredients in bread. Food Anal. Methods.

[57] Saputra I., Jaswir I., Akmeliawati R. (2018). Identification of pig adulterant in mixture of fat samples and selected foods based on FTIR-PCA wavelength biomarker profile. Int. J. Adv. Sci. Eng. Inf. Technol..

[58] Salleh N.A.M., Hassan M.S., Jumal J., Harun F.W., Jaafar M.Z. (2018). Differentiation of edible fats from selected sources after heating treatments using fourier transform infrared spectroscopy (FTIR) and multivariate analysis. Proceedings of the AIP Conference Proceedings.

[59] Ahda M., Safitri A. (2016). Development of lard detection in crude palm oil (CPO) using ftir combined with chemometrics analysis. Int. J. Pharm. Pharm. Sci..

[60] Upadhyay N., Jaiswal P., Jha S.N. (2018). Application of attenuated total reflectance Fourier Transform Infrared spectroscopy (ATR–FTIR) in MIR range coupled with chemometrics for detection of pig body fat in pure ghee (heat clarified milk fat). J. Mol. Struct..

[61] Alkhalf M.I., Mirghani M.E.S., Nazrim Marikkar J.M., Hammed A.M., Kabbashi N.A. (2017). The use of analytical techniques for qualitative differentiation of lipids extracted from cheese samples and lard. J. Food Agric. Environ..

[62] Waskitho D., Lukitaningsih E., Sudjadi, Rohman A. (2016). Analysis of lard in lipstick formulation using FTIR spectroscopy and multivariate calibration: A comparison of three extraction methods. J. Oleo Sci..

[63] Komponen P., Lemak B., Daripada B., Haiwan L., Nina Naquiah A.N., Marikkar J.M.N., Mirghani M.E.S., Nurrulhidayah A.F., Yanty N.A.M. (2017). Differentiation of fractionated components of lard from other animal fats using different analytical techniques. Sains Malays..

[64] Rohman A., Himawati A., Triyana K., Sismindari, Fatimah S. (2017). Identification of pork in beef meatballs using Fourier transform infrared spectrophotometry and real-time polymerase chain reaction. Int. J. Food Prop..

[65] Rahayu W.S., Martono S., Sudjadi, Rohman A. (2018). The potential use of infrared spectroscopy and multivariate analysis for differentiation of beef meatball from dog meat for Halal authentication analysis. J. Adv. Vet. Anim. Res..

[66] Rahayu W.S., Rohman A., Martono S., Sudjadi S. (2018). Application of FTIR spectroscopy and chemometrics for halal authentication of beef meatball adulterated with dog meat. Indones. J. Chem..

[67] Cebi N., Dogan C.E., Mese A.E., Ozdemir D., Arıcı M., Sagdic O. (2019). A rapid ATR-FTIR spectroscopic method for classification of gelatin gummy candies in relation to the gelatin source. Food Chem..

[68] Hashim D.M., Man Y.B.C., Norakasha R., Shuhaimi M., Salmah Y., Syahariza Z.A. (2010). Potential use of Fourier transform infrared spectroscopy for differentiation of bovine and porcine gelatins. Food Chem..

[69] Lee J.Y., Park J.H., Mun H., Shim W.B., Lim S.H., Kim M.G. (2018). Quantitative analysis of lard in animal fat mixture using visible Raman spectroscopy. Food Chem..

[70] Nedeljkovic A., Tomasevic I., Miocinovic J., Pudja P. (2017). Feasibility of discrimination of dairy creams and cream-like analogues using Raman spectroscopy and chemometric analysis. Food Chem..

[71] Berhe D.T., Eskildsen C.E., Lametsch R., Hviid M.S., van den Berg F., Engelsen S.B. (2016). Prediction of total fatty acid parameters and individual fatty acids in pork backfat using Raman spectroscopy and chemometrics: Understanding the cage of covariance between highly correlated fat parameters. Meat Sci..

[72] Boyaci I.H., Uysal R.S., Temiz T., Shendi E.G., Yadegari R.J., Rishkan M.M., Velioglu H.M., Tamer U., Ozay D.S., Vural H. (2014). A rapid method for determination of the origin of meat and meat products based on the extracted fat spectra by using of Raman spectroscopy and chemometric method. Eur. Food Res. Technol..

[73] Lambert I.A., Kokini J.L. (2001). Effect of L-cysteine on the rheological properties of wheat flour. Cereal Chem..

[74] Cebi N., Dogan C.E., Develioglu A., Yayla M.E.A., Sagdic O. (2017). Detection of L-Cysteine in wheat flour by Raman microspectroscopy combined chemometrics of HCA and PCA. Food Chem..

[75] Hatzakis E., Dais P. (2008). Determination of water content in olive oil by ^31^ P NMR spectroscopy. J. Agric. Food Chem..

[76] Fadzillah N.A., Man Y.B.C., Rohman A., Rosman A.S., Ismail A., Mustafa S., Khatib A. (2015). Detection of butter adulteration with lard by employing H-NMR spectroscopy and multivariate data analysis. J. Oleo Sci..

[77] Otte D.A.L., Borchmann D.E., Lin C., Weck M., Woerpel K.A. (2014). ^13^C NMR spectroscopy for the quantitative determination of compound ratios and polymer end groups. Org. Lett..

[78] Fadzillah N.A., Rohman A., Salleh R.A., Amin I., Shuhaimi M., Farahwahida M.Y., Rashidi O., Aizat J.M., Khatib A. (2017). Authentication of butter from lard adulteration using high-resolution of nuclear magnetic resonance spectroscopy and high-performance liquid chromatography. Int. J. Food Prop..

[79] Derewiaka D., Sosińska E., Obiedziński M., Krogulec A., Czaplicki S. (2011). Determination of the adulteration of butter. Eur. J. Lipid Sci. Technol..

[80] Sacchi R., Paduano A., Caporaso N., Picariello G., Romano R., Addeo F. (2018). Assessment of milk fat content in fat blends by ^13^C NMR spectroscopy analysis of butyrate. Food Control.

[81] Keire D., Mulloy B., Chase C., Al-Hakim A., Cairatti D., Gray E., Hogwood J., Morris T.S., Mourao P., Soares M.D.L.C. (2015). Diversifying the global heparin supply chain: Reintroduction of bovine heparin in the United States?. Pharm. Technol..

[82] Ouyang Y., Han X., Yu Y., Chen J., Fu L., Zhang F., Linhardt R.J., Fareed J., Hoppensteadt D., Jeske W. (2019). Chemometric analysis of porcine, bovine and ovine heparins. J. Pharm. Biomed. Anal..

